# Is varicocele repair justified in infertile men with clinical varicocele, normal conventional semen parameters and elevated sperm DNA fragmentation (SDF)?

**DOI:** 10.1080/20905998.2025.2494180

**Published:** 2025-04-21

**Authors:** Khaled Almekaty, Ahmed Ghaith, Maged Ragab, Haitham Elbardisi

**Affiliations:** a Urology Department, Tanta University, Tanta, Egypt; b Urology Department, Hamad Medical Corporation, Doha, Qatar; c Clinical Urology, Weill Cornell, Doha, Qatar; d Urology, College of Medicine, Qatar University, Doha, Qatar

**Keywords:** Varicocele, sperm DNA fragmentation, SDF

## Abstract

Varicocele is a prevalent cause of male infertility, with sperm DNA fragmentation (SDF) identified as a key factor. Although varicocele repair (VR) has been associated with improved fertility outcomes, its effectiveness in men who have a palpable varicocele, normal conventional semen analysis, and high SDF levels is still not fully understood. This review summarizes existing evidence regarding the connection between varicocele and SDF, the effect of VR on both SDF and fertility outcomes and focuses on infertile men experiencing these specific conditions. Current literature indicates that VR can significantly improve SDF and reproductive success following surgery, independent of the surgical approach, SDF testing method, or preoperative SDF levels. Nonetheless, additional research is necessary to validate these results and to establish the best management strategies for these individuals. We recommend that organizations engaged in the area of male infertility take into account the significance of SDF testing in the decision-making process concerning VR, particularly for men who present with a palpable varicocele and demonstrate normal findings in conventional semen analysis.

## Introduction

Each heterosexual couple with regular unprotected sexual intercourse for more than a year has a 10% chance of infertility. Estimates of 48 million couples suffer from infertility worldwide. This can be attributed to a male factor in 30% of cases [1]. A broad spectrum of possible aetiologies can lead to male factor infertility, of which varicocele is of particular importance, being prevalent and surgically correctable [1].

Varicocele is found in 15% of men and considered the aetiological factor of infertility in 35% to 44% of men with primary and 45% to 80% of men with secondary infertility [2].

Varicocele has always been a debatable topic regarding its role in the pathophysiology of male infertility and the impact of varicocele repair (VR) on sperm parameters and fertility outcomes. However, there is accumulating strong evidence nowadays that supports a causal relationship between varicocele and male infertility and proves a positive impact of VR on fertility outcomes, whether through natural pregnancy or Assisted Reproduction Techniques (ART) [3].

Varicocele can lead to infertility through multiple mechanisms such as impaired venous drainage with elevation of Reactive Oxygen Species (ROS) levels, mounting scrotal temperature, accumulation of metabolites, testicular hypoxia and immunological effect. Eventually, these mechanisms will lead to increased oxidative stress (OS) and consequently impaired spermatogenesis and defective sperm fertilization abilities [4].

Clinical varicocele was identified in 25.4% of men with abnormal semen analysis and 11.7% of those with normal semen analysis [5]. Those with normal semen analysis are classified as having unexplained infertility. According to Simon et al., 87% of these men exhibit a high SDF of over 25%, which is the threshold he used to differentiate between infertile and fertile men [6]. Approximately 50% of men with clinically palpable varicocele have elevated SDF levels, and VR is associated with a reduction in SDF, particularly in cases of higher-grade varicoceles [7]. However, the literature lacks information on the proportion of men with high SDF among those who have normal semen analyses and clinically palpable varicoceles.

Recently, the literature has increasingly addressed the importance of SDF in the aetiopathogenesis of male infertility, including those with palpable varicocele. However, the guidelines lack recommendations regarding the value of VR in patients with palpable varicoceles, normal conventional semen analysis, and high SDF. So, this review aimed to explore the up-to-date available evidence on varicocele and SDF and the effect of VR on SDF and consequently on fertility outcomes that may support or refute VR in the cohort of patients mentioned above.

## Methods and evidence acquisition

A thorough literature search was conducted using Scopus and PubMed. Our search terms were varicocele AND sperm DNA fragmentation AND SDF. We looked for studies published in English from the inception to the present. We included original, retrospective, and prospective studies with semen and SDF before and after VR. We excluded case reports, book chapters, systematic reviews, meta-analyses, and reports with no semen analysis or SDF testing before and after VR. We screened the titles and abstracts of all search results to ensure their relevance to our selection criteria. The authors, year, study design, sample size, number of patients, primary or secondary infertility, duration of infertility, method of VR, semen analysis and SDF prior and after VR and methods of SDF testing have all been included in a spreadsheet. The data was divided among 2 researchers and further assessed and revised by a reviewer for accurate data extraction.

## Search results and discussion

Using the aforementioned search strategy, 74 potentially relevant abstracts were extracted. Further exclusion of review articles, case reports, book chapters, papers on irrelevant topics, and incomplete text articles was done. Finally, **23 manuscripts** were found to fit the inclusion criteria accurately. Table 1 summarizes the findings of these studies.

**Table 1.** Trials included in our study.

	Study	Design	Number of participants	Type of surgery	Type of SDF testing	Pre SDF	Post SDF	P value
1	Smit et al. [7]	Observational	31a	Unspecified	Sperm chromatin dispersion assay	35.3 ± 14.3	28.6 ±14.7	*P = 0.019*
2	Nasr Esfahani et al. [8]	Observational	70	Unspecified	Sperm chromatin dispersion assay	45.69 ± 2.43	35.9 ± 2.4	*P=0.01*
3	Dada et al. [9]	Observational	11	Unspecified	Comet assay	60.82 ± 7.12	32.58 ± 6.12	*Unspecified*
4	Ghazi et al. [10]	Observational	82	Microsurgery	TUNEL assay	21.5 ± 11.2	13 ± 14.3	*P < 0.001*
5	Zini et al. [11]	Observational	25	Microsurgery	Sperm chromatin dispersion assay	18 ± 11	11 ± 6	*P=0.0009*
6	Sadek et al. [12]	Observational	72	Non microsurgery	Sperm Chromatin Aniline Blue Staining	65.15 ± 20.97	51.9 ± 15.2	*P <.001*
7	La Vignera et al. [13]	Observational	30	Microsurgery	TUNEL assay	5 ± 3	2.1 ± 0.4	*P < 0.05*
8	Gabriel et al. [14]	Observational	14	Microsurgery	Sperm chromatin dispersion assay	22.4 ± 11.9	12.6 ± 6	*P= 0.002*
9	Li et al. [15]	Observational	19	Microsurgery	Sperm chromatin dispersion assay	28.4 ± 15.6	22.4 ± 12.9	*P= 0.018*
10	Smit et al. [16]	Observational	49	Unspecified	Sperm chromatin dispersion assay	35.2 ± 13.1	30.2± 14.7	*P = 0.019*
11	Mohammed et al. [17]	Observational	75	Non microsurgery	Acridine Orange (AO) Assay	32.4 ± 7.4	20 ± 4.1	*P= 0.05*
12	Tavalaee et al. [18]	Observational	23	Unspecified	Unspecified	15.9 ± 1.2	10.8 ± 1.1	*P < 0.001*
13	Telli et al. [19]	Observational	72	Non microsurgery	Acridine Orange Test (AOT)	34.5 ± 3.3	28.2 ± 3.5	*P = 0.024*
14	Ni et al. [20]	Observational	51 patients (G I: 19 patients G II: 18 patients G III: 14 patients)	Microsurgery	Sperm chromatin dispersion assay	G I: 23.56 ± 7.55 G II: 27.75 ± 9.05 G III: 30.03 ± 8.27	G I: 19.54 ± 5.48 G II: 22.38 ± 4.54 G III: 21.82 ± 5.95	*P > 0.05*
15	Abdelbaki et al. [21]	Observational	55	Microsurgery	Sperm chromatin dispersion assay	29.49 ± 8.58	18.78 ± 7.23	*P < 0.001*
16	Zaazaa et al. [22]	RCT	120 (40 in group I, 40 in group II, and 40 in group III)	Microsurgery	Sperm chromatin dispersion assay	Group I: 34.6 ± 4.1 Group II: 33.4 ± 2.3 Group III: 34.3 ± 2.9	Group I: 28.3 ± 5.2 Group II: 27.8 ± 3.9 Group III: 25.1 ± 4.2	*P < 0.05*
17	Sun et al. [23]	Observational	358 (179 underwent bilateral, 179 underwent unilateral VR)	Microsurgery	Unspecified	21.6 ± 7.1	11.8 ± 6	*P < 0.05*
18	Vahidi et al. [24]	Observational	30	Microsurgery	TUNEL	15.93 ± 4.96	10.86 ± 4.44	*P=0.001*
19	Afsin et al. [25]	Observational	40	Unspecified	TUNEL	20.57 ± 4.6	15.3 ± 3.63	*P< 0.001*
20	Camargo et al. [26]	Observational	25	Microsurgery	Unspecified	43.4 ± 22.62	32.9 ± 8.87	*P= 0.091*
21	Abbasi et al. [27]	Observational	22	Microsurgery	Sperm chromatin dispersion assay	13.65 ± 11.44	10.38 ± 4.64	*P ≤ 0.02*
22	Fathi et al. [28]	Observational	95 (45 in study group, 50 in control group)	Microsurgery	Sperm chromatin dispersion assay	Study group: 34.93 ± 5.56 Control group: 35.33 ± 6.12	Study group: 25.75 ± 5.15 Control group: 31.26 ± 5.34	*P < 0.001*
23	Kavoussi et al. [29]	Observational	141 (20 in TA, 121 in NTA)	Microsurgery	Sperm chromatin dispersion assay	TA: 29.7 ± 5.0 NTA: 35.3 ± 11.6	TA: 22 ± 0 NTA: 19.6 ± 5.3	*P= 0.0001*

TA: testicular atrophy, NTA: non testicular atrophy.

### SDF and its effect on fertility outcomes

According to recent data, high SDF is associated with poor reproductive outcomes for natural conception and ART. Males with elevated SDF have lower natural pregnancy rates compared to normal controls [30–32]. Furthermore, elevated SDF is linked to miscarriage. In a systematic review and meta-analysis of couples suffering from recurrent idiopathic miscarriage, SDF levels were found to be higher as compared to fertile controls (*p* < 0.001) [33]. Moreover, regarding ART, elevated SDF has been linked to recurrent pregnancy loss and lower IUI and IVF success rates; however, the effect on ICSI outcome is controversial.

The molecular mechanisms by which high SDF negatively affects male fertility are not fully understood [5]. Studies, however, have consistently shown that SDF is primarily induced by two main mechanisms: defective maturation and abortive apoptosis occurring within the testis, or oxidative stress (OS) throughout the male reproductive tract [34] During spermatogenesis, immature sperm cells undergo a series of complex processes to mature into functional spermatozoa. However, when this process is disturbed, immature sperm cells may become apoptotic, leading to the formation of fragmented DNA. This can occur due to various factors, including genetic mutations, environmental toxins, and hormonal imbalances. As a result, the sperm cells that manage to mature may possess damaged DNA, which can compromise their fertilization potential [35].

Elevated SDF can initiate the activation of caspases, leading to sperm DNA degradation and cytochrome C release, which further promote apoptosis. Additionally, elevated SDF can impact the epigenetic alterations of sperm DNA, which are changes in gene expression that do not involve modifying the DNA sequence itself. DNA methylation and histone modifications are examples of epigenetic changes that are critical for controlling gene expression and can influence embryonic development and the well-being of the offspring [36].

Moreover, OS can indirectly induce SDF through by-products of lipid peroxidation, particularly malondialdehyde (MDA) and 4-hydroxynonenal (4HNE). These mechanisms can result in lower sperm fertilization potential during natural pregnancy or ART [37].

The potential utility of SDF testing in clinical practice depends on its predictive value for achieving natural pregnancy and the success of assisted reproduction [38]. Currently, the accepted indications for SDF testing are recurrent pregnancy loss, recurrent ART failure, exposure to gonadotoxins, ageing and unexplained infertility. Relative indications might include patient selection for VR, deciding on the most appropriate ART and those with modifiable lifestyle factors [39].

### SDF in varicocele patients and the pathophysiological link

There is growing evidence indicating that varicocele is associated with elevated SDF levels, and VR could have a beneficial impact on SDF [40]. Herein, we summarize the current evidence on how varicocele can lead to high SDF and, consequently, lower natural pregnancy and ART outcomes. Varicocele is believed to contribute to SDF through a multifaceted mechanism involving OS, hyperthermia, impaired testicular function, and inflammation. The abnormal dilation of the scrotal veins in varicocele can lead to venous stasis and hypoxia and, eventually, the production of ROS, such as superoxide anion, hydroxyl radical, and hydrogen peroxide, which can damage sperm DNA by inducing strand breaks, base modifications, and chromatin cross-linking. This can ultimately result in SDF [41]. Furthermore, varicocele associated scrotal hyperthermia can impair sperm DNA integrity by denaturing proteins, disrupting chromatin structure, and activating DNA repair pathways that can introduce errors in the genetic material [42]. Additionally, varicocele can disrupt testicular function, leading to defects in sperm chromatin condensation, DNA packaging, and epigenetic regulation, which can further contribute to SDF [35]. Finally, the inflammatory response triggered by varicocele can lead to the production of pro-inflammatory cytokines, such as interleukin-1 beta (IL-1β), tumor necrosis factor-alpha (TNF-α), and interleukin-6 (IL-6), which can damage sperm DNA by activating OS pathways, inducing DNA damage and disrupting sperm function [43].

### The impact of VR on SDF and fertility outcome

The guidelines of the American Urological Association/American Society for Reproductive Medicine and the European Association of Urology recommend VR in patients with palpable varicoceles and abnormal semen parameters [44,45]. VR is clearly associated with improvement in semen parameters, with 60%–68% of patients exhibiting improvements in semen parameters 6 months after surgery [46].

In this review, a total of 23 studies addressed the effect of VR on SDF and, consequently, fertility outcomes. Table 1 summarizes the findings of these studies. The whole 23 studies showed that VR significantly reduces the level of SDF.

A meta-analysis by Cannarella et al. included a total of 29 studies; 23 reporting on SDF after VR and 6 reporting on seminal malondialdehyde. They concluded that VR significantly improves SDF, independent of the type of surgical technique (microsurgical versus non-microsurgical), type of SDF test or preoperative SDF levels [47]. Similar findings were encountered in another systematic review and meta analysis, including 15 studies [6].

Concerning patients undergoing ART, the results of a recent meta-analysis (in which 16 cohort studies with a total of 3,106 couples were included) showed a significant decrease in pregnancy rates using sperm with high DNA damage in IVF cycles. In contrast, there was no difference in pregnancy rates in ICSI cycles [48]. On the other hand, Esteves et al. conducted a systematic review and meta-analysis which included 4 articles; and divided patients into 2 groups: group 1 underwent VR then ICSI, while group 2 underwent ICSI without repair of their varicoceles. They concluded that performing VR in patients with clinical varicocele before ICSI is associated with improved pregnancy outcomes [49].

### SDF testing in patients with unexplained infertility

Several guidelines recommend SDF testing in the setting of unexplained infertility. Unexplained infertility is defined as the inability to conceive despite normal male and female evaluation and investigations. More than 30% of this patient population was found to have elevated SDF [34].

Furthermore, 15–20% of the normal populations have varicoceles [47]. The majority are asymptomatic and have normal semen analysis. At the same time, patients with normal conventional semen analysis and varicocele might have elevated SDF as shown in the previous paragraphs. Therefore, testing these patients can help to assess their fertility potentials and affect their reproductive outcome.

### VR in patients with isolated elevated SDF

Available guidelines recommend VR in the setting of palpable varicoceles in infertile men with abnormal semen parameters. There is a lack in the literature and, hence, the guidelines statement addressing the specific scenario of whether VR should be offered to men with elevated SDF and normal semen parameters [45].

Surprisingly, despite extensive research on varicocele and its impact on male fertility, there is a noticeable lack of studies addressing the outcomes of VR in a specific subset of patients: those with palpable varicocele, normal conventional semen parameters and elevated SDF. Only one pilot study examined this specific research point. This scarcity can be attributed to the consideration of abnormal semen parameters as an indication for VR, leading to the exclusion of patients with normal semen from such studies. Fathi et al. conducted a pilot study in 2021 that included this specific cohort of patients. Microsurgical subinguinal VR was performed on 45 cases (study group), while 40 had no intervention (controls). Six months after VR, the mean SDF was reduced in both groups, with this reduction being statistically greater in the study group (*p* < 0.001). After one year, the natural pregnancy rate was 62% in the VR group compared to only 30% in the control group (*p* = 0.009). They concluded that VR has a positive impact on SDF and natural pregnancy rates in this specific cohort of patients with infertility, palpable varicoceles, normal semen parameters and high SDF.

This scenario represents a critical gap in the literature, as current studies primarily focus on VR in men with abnormal semen parameters or clinical varicocele without consideration of sperm DNA integrity. Even though there is a clear association between varicocele and SDF and the established effect of SDF on natural and ART pregnancy outcomes. The significance of this gap lies in the growing recognition of SDF as an independent marker of male infertility, influencing reproductive outcomes beyond traditional semen analysis parameters. Addressing this gap requires targeted research efforts to elucidate whether VR can effectively improve high SDF levels and fertility outcomes in this specific patient subgroup. Future studies should employ rigorous methodologies, including standardized assessments of sperm DNA integrity and long-term follow-up, to assess the clinical relevance of VR in improving fertility potentials in these patients. Such research endeavours are crucial for optimizing clinical management strategies and providing evidence-based recommendations for personalized treatment approaches in male infertility.

### SWOT analysis of the study

#### Strengths

The article provides a comprehensive review of the current literature on SDF in varicocele patients, explicitly focusing on the cohort of patients with palpable varicocele, normal conventional semen analysis, and high SDF. It also includes a detailed discussion of SDF’s molecular mechanisms and risk factors.

The article reviewed the studies evaluating the association between varicocele and SDF and the impact of VR on SDF and fertility outcomes.

#### Weaknesses

Unfortunately, 3 points remain as weaknesses even after conducting this narrative review. The first is the best SDF test and its optimal cut-off point. The second is the indication of when to do the test. The last point of weakness is the rarity of well-designed studies addressing the specific scenario of the effect of VR on patients with palpable varicocele, normal conventional semen analysis and high SDF. The first weak point was tackled by Agarwal et al., who tried to sort out the first two points and recommended SDF testing in men with unexplained, idiopathic male infertility, repeated miscarriage, lifestyle risk factors, varicocele and repeated ART failure (Grade C recommendation – low quality of evidence). Also, they recommended the TUNEL test as the most reliable and accurate test, but the vast heterogeneity of the published data made clear cut-off values challenging to conclude. Esteves et al. provided a review of all the pros and cons of different SDF tests but without making any preferences. Also, they proposed similar indications for offering SDF testing (level of evidence B-D – moderate- very low quality of evidence). Also, they recommended the test for men undergoing sperm cryopreservation (Grade D recommendation).

#### Opportunities

The article can be used as a reference for clinicians and researchers in the field of male infertility. The article can be used to guide clinical decision-making, especially in the specific scenario of male infertility with palpable varicocele, apparently normal semen analysis with high SDF. The article can be used to identify areas for future research and investigation, especially in the field of SDF and its effect on natural and assisted pregnancy, identify subgroups that may benefit from SDF testing, and encourage a personalized patient management approach.

#### Threats

What exceeds its limit will turn against itself. So, the addition of SDF testing in the 6th edition of the WHO manual (2021) may result in its inappropriate use without proper justification. Certain techniques for assessing SDF necessitate costly equipment and specialized personnel, potentially increasing the financial burden of diagnosing and managing the already costly male infertility problem. Unnecessary or excessive utilization of SDF testing and antioxidants may cause reductive stress as a negative consequence.

## Conclusion

This narrative review presents evidence of the association between varicocele and elevated SDF. VR significantly improves SDF and, consequently, reproductive outcomes. Improvement of SDF after VR is independent of the type of surgical technique, type of SDF test, or preoperative SDF baseline value.

The gap in the literature is the absence of well-designed studies addressing clear recommendations regarding the indications of SDF testing and the optimization of its clinical utility, especially in men with palpable varicocele and normal conventional semen analysis and how to use SDF testing to justify VR in this particular cohort of patients.

### Recommendation

The hypothesis is that patients with unexplained infertility and clinical varicocele must be evaluated with SDF for better patient counselling regarding their options of treatment, including VR or ART. More studies investigating the use of more qualitative tools as SDF rather than only quantitative ones as conventional semen analysis are needed in the decision-making process of VR.
